# The Military Surgery of To-Day

**Published:** 1915-10-02

**Authors:** 


### THE INSTITUTIONAL LIBRARY.

### THE MILITARY SURGERY OF TO-DAY.

Since the war began a large number of new problems in treatment—many of them old and forgotten problems newly come to light and susceptible of solution by varied and diverse principles of modern medicine and surgery—have forced themselves upon the attention of those whose task it is to restore our sick and wounded soldiers to health. In a general way it is true, as Sir Almroth Wright says in a most interesting book recently published,* that nearly the whole care of the sick and wounded has fallen to the civil practitioners enlisted for temporary service with the R.A.M.C. He goes on to point out that in military hospitals abroad the practitioner is confronted with wholly unfamiliar problems in connection with projectile wounds and wound infections; he has to take immediate action; he has very little opportunity of finding out what has happened in similar cases or of seeing the final results of his work, and thus learning whether his treatment is right or wrong. The military hospitals in France, says this authority, have become little more than clearing hospitals. This statement is often made, but is only partially true; doubtless it is truer of some hospitals or groups of hospitals than of others, depending on the views of the superior authorities exercising jurisdiction over them. At any rate, when base hospitals are administered too much on the lines of clearing stations, it is undeniable that some of the evils which Sir Almroth notes are fostered; one remedy, which he does not consider, is to administer them less on the clearing station pattern, and though this may at exceptional times be impossible on grounds of military necessity, it is not so except in very rare and occasional emergencies.

Sir A. Wright's escape from the dilemma is to make a fundamental change in the organisation of the Medical Service; that is, to break away from the principle of free arbitrament in treatment for the medical officer, and to provide that all treatment shall be regulated by orders and instructions. This large issue involves a direct conflict between the cherished professional tradition that every man is quite unfettered in his choice of treatment, and the foundation principle of the Army that every man shall work, not as he individually thinks best, but as part and parcel of a great machine. Sir Almroth buttresses his advocacy of the latter system by adducing the marvellous results of anti-typhoid inoculation and of anti-tetanus serum (preventive), both of which are enjoined by direct instruction from headquarters.

In this trenchant criticism two contrasted systems are outlined in bold strokes, but as a matter of fact there is a middle course which, with our national habit of compromise, we have already adopted to a considerable extent. Attractive as Sir A. Wright's panacea sounds, there are certain serious objections to it. It centralises things too much, would tend to paralyse individual resource and initiative, and would lay too much responsibility and repose too much power in the hands of the person nominated in the scheme as "Professional Head to the Service for the Treatment of the Sick and Wounded." He who could fulfil the requirements for such a post would be a giant in intellect indeed, and if anyone less than superhuman were entrusted with it the results might be disastrous. It is not possible, in our judgment, so to standardise treatment that a rule-of-thumb routine can be applied to every case the moment the diagnosis is made; and although Sir Almroth may reply that he does not suggest such a thing, yet that would in practice be the result of the system he proposes. For responsibility is the keystone of successful treatment, and his system would abolish responsibility.

It has been said that there is a middle course between the plan of leaving each medical officer to work out his own methods of treatment, and to fall into the same errors and pitfalls as many others before him. To some extent the presence of a recognised "surgical specialist" (with extra pay as well as the prestige of the name) at each general hospital (in France) is a valuable educative help to the other medical officers. This officer is usually a temporarily commissioned civilian, and the holder of one of the higher surgical diplomas. His advice is frequently sought and acted on by those of less experience, both by way of actual bedside consultation and of the "shop" that is talked in medical messes from morning till night. The leaven of such a specialist's influence spreads amazingly quickly through the wards of a hospital, especially when (as is usually the case) the ward officers are comparatively young men at a receptive time of life. Again, the surgical specialist himself and, in fact, all the officers entrusted with surgery in any given hospital, are kept in fairly close touch with new developments and with the experience gained in other hospitals by the visits of the consulting surgeons. There are a number of eminent and experienced surgeons whose functions are to supervise each a group of hospitals and to exercise an advisory capacity therein. To the energy and tact with which this work is done there is a general consensus of testimony among recently returned R.A.M.C. officers. In at least one large centre a local medical society was long ago formed, at which papers on war surgery are read and discussions take place with the happiest results. Printed pamphlets composed by the consulting surgeons (and physicians) on particular topics are frequently distributed to all medical officers under their guidance, and actual demonstration by the visits is, of course, an important part of the method.

Here there is a picture somewhat different from that which Sir Almroth draws. The lights are not

* *Wound Infections.* By Sir Almroth E. Wright, M.D., F.R.S. University of London Press. Published by Hodder and Stoughton. Pp. 96. Price 2*s.* 6*d.* net.

quite so strong nor the shadows so deep as in the two contrasted sketches which he lays before the world. Nor can Sir Almroth's model scheme of centralised despotism in regard to medical and surgical treatment be regarded altogether apart from those views on wound treatment which he has propounded; one cannot avoid the notion that it is *his* views on the best methods of surgery that he would like to see universally enforced. Beautiful as his experimental data always are, and brilliant as is the reasoning he builds upon them, it is impossible to overlook this fact: that many able surgeons in France who have tried some of his ingenious and novel methods of treatment conscientiously and thoroughly are not satisfied that its results are always better (if as good) as other kinds of treatment; nor can the profession forget that Sir Almroth's fertile genius has on previous occasions led him astray. The fatal objections to his plan of autocratically controlled medical treatment are the impossibility of finding an infallible autocrat and the deteriorating effect upon any man's scientific personality of granting him autocratic powers. At the same time, while refusing assent to the conclusion of his argument, it is but just to add a tribute to the originality of many of the ideas disclosed in this book and to the energy with which he prosecutes research and the earnestness with which he endeavours to think and speak clearly.

The British Journal of Surgery. Vol. III., No. 9. July 1915. (Bristol: J. Wright and Sons.)

Though somewhat delayed by the military censorship regulations concerning certain contributions dealing with war surgery, the midsummer issue of the *British Journal of Surgery* is by no means merely a collection of experiences from the war hospitals; indeed, civilian surgery of the ordinary type occupies much the greater part of its pages. The longest and perhaps, from a scientific standpoint, the most satisfactory of the contributions is that of Mr. R. B. Carslaw upon the significance of peritoneal exudates. He concludes as a result of a painstaking research: (1) That the "mononuclear phagocytes" in peritoneal exudate consist of two different types of cell, endothelial cells and large mononuclear leucocytes, which not only have two distinct sources of origin, but also can easily be distinguished from each other in all stages of their functional activity by the "indo-phenol synthesis" test for oxydase. (2) That examination of the peritoneal exudate is of great prognostic value in cases of spreading peritonitis, as it indicates the amount and virulence of the bacterial invasion and the extent to which the reactive forces of the peritoneal cavity have been or are being developed. . . . (3) That the contention that an immediate examination of the exudate in cases of spreading peritonitis supplies information which indicates whether drainage of the peritoneal cavity is necessary . . . must be modified by three factors: (*a*) local drainage is often necessary, although drainage of the general peritoneal cavity may be not only unnecessary, but also injudicious; (*b*) drainage of the general peritoneal cavity is only called for in very exceptional cases; (*c*) *effective* drainage of the peritoneal cavity is impossible. Then Dr. G. E. Armstrong, of Montreal, publishes some interesting statistics from his large experience of breast cancer operations. Weighting his figures against himself to the utmost, he produces 30 per cent. of patients alive and well without recurrence three years or more after operation. The papers on military surgery are a little disappointing, if one excepts a careful essay by Mr. C. A. Joll on gunshot wounds of the skull; the *Journal of the Royal Army Medical Corps* intercepts a good many papers that might otherwise reach the *British Journal of Surgery*. In the section reserved for rare and obscure cases is an account of a most extraordinary suicide by a man who thrust a red-hot poker completely through his chest from front to back. The patient lived for eighteen days, and for part of the time his condition gave rise to comparatively little anxiety. This amazing case deserves a niche in the text-books of medical jurisprudence.

A Hospital Handbook in English and French. By H. Meugens. (London: Simpkin, Marshall, Hamilton, Kent and Co., Ltd. Price 1s.)

This dictionary is of a convenient size for the pocket, and contains a very large number of words and phrases of the kind that would be required by those serving in France. The vocabulary, which includes a great variety of words and phrases, is divided into two parts, namely, (1) drugs and dressings; (2) medical terms, nursing and other useful phrases. The latter section, which fills quite nine-tenths of the book, is naturally a heterogeneous list of words and expressions; motoring and medicine, warfare and workshop all contribute their quota. The words and phrases are arranged alphabetically, and not elaborately sorted, though, following a key-word, are given useful sentences and compound words. The dictionary is solely English-French, but is eminently practical, and should prove of considerable utility to those who are working in hospitals in France.

Measures for Avoidance and Extermination of Flies, Mosquitoes, Lice, and other Vermin. By H. Maxwell-Lefroy, M.A., F.Z.S. Copyright 1915. (Privately printed.)

Knowledge of the power for ill possessed by flies and other insects is being at present freely disseminated, but inquiries made seem to show that the general public is not well instructed on the subject of preventive measures, on the life-history of the insects, and their efficiency as germ-carriers. For this reason a handy pamphlet, which has just been prepared by Mr. Maxwell-Lefroy, will be regarded as a practical boon by doctors, nurses, and other workers who are striving to minimise the harm done to mankind by these small pests. The pamphlet has been produced at the expense of friends, and is apparently not for sale. Anyone desiring a copy for personal use, or to send abroad, can obtain it on application to Miss E. Chick, Chestergate, Ealing, W., who has undertaken the duties of hon. secretary and treasurer to this enterprise. In some sixteen pages there are given plain and practical instructions for dealing with flies, lice, fleas, mosquitoes, and bugs. Flies receive the most attention, and two excellent fly-traps are described and illustrated. At the end are given useful formulæ and directions for preparing the insecticides mentioned in the text, among which the fly poisons and baits hold easily the first place. The poisons recommended are formalin in dilute solution and the combination of arsenite of soda with treacly liquids. The author of the pamphlet is professor of entomology at the Imperial College of Science, South Kensington; he has been assisted by Dr. Harriette Chick, of the Lister Institute.

